# Indian Real-World Data on Acute Cough Evaluation Through Cough Sounds and Auscultation Findings and Its Complications

**DOI:** 10.7759/cureus.109902

**Published:** 2026-05-29

**Authors:** Surinder Jindal, Agam Vora, Harshad Malve, Someshwar Rayasam, Nikhil Bangale

**Affiliations:** 1 Pulmonary Medicine, Jindal Chest Clinic, Chandigarh, IND; 2 Pulmonology, Vora Clinic, Mumbai, IND; 3 Medical Safety Sciences, Kenvue, Mumbai, IND

**Keywords:** acute cough, auscultations, cough acoustics, cough sounds, diagnosis

## Abstract

Introduction

The evaluation of acute cough in primary care settings remains inconsistent, and cough sounds are seldom used for evaluation. This retrospective observational study examined how clinicians across different medical specialties utilize cough sounds and auscultations alongside different diagnostic approaches for the evaluation and categorization of acute cough and overall acute cough-related complications.

Methods

Electronic medical records from 2017 to 2023 for patients with acute cough were analyzed retrospectively across pediatric (<18 years), adult (18-65 years), and elderly (>65 years) age groups. Auscultation patterns, diagnostic investigations, and cough-related complications were analyzed.

Results

Unspecified cough predominated across all age groups - pediatric: 128,184 (88.22%); adult: 707,788 (78.29%); and elderly: 154,129 (76.48%). The most common auscultatory finding was wheezing reported by pulmonologists (376, 6.73%) in pediatric patients, 2,954 (5.53%) in adults, and 934 (6.45%) in the elderly. Chest X-ray was the primary diagnostic tool across specialties, whereas imaging use was conservative in pediatric populations (3,767, 15.7%), 33,850 (34.9%) in adults, and 7,869 (56.4%) in the elderly. Chest pain emerged as the most predominant complication in 1,631 (6.78%) pediatric patients, 22,906 (23.61%) adults, and 5,095 (36.58%) elderly. Most general practitioners demonstrated nominal use of cough sounds and auscultatory findings for the evaluation of cough, as it is not a regular practice.

Conclusion

The use of cough sounds and auscultations remains underutilized in practice, especially by general practitioners. Incorporating systematic acoustic analysis, including cough sounds and auscultatory patterns in primary care, could improve acute cough categorization, reduce or better plan diagnostic testing, and enable targeted and prompt treatment. Future research should focus on developing standardized tools and training programs to optimize acute cough management.

## Introduction

Cough is a fundamental airway defense reflex characterized by a forceful expulsion of air triggered by mechanical or chemical stimulation of the respiratory tract [1,2]. Depending on the duration, cough can be acute (lasting for <3 weeks), subacute (lasting for three to eight weeks), and chronic (lasting for >8 weeks) [2]. As reported in 2023, the global prevalence of cough is 9.6%, with a substantial burden of 5%-10% reported in India [3,4]. As reported in 2023, an average Indian adult experiences three episodes of cough per year, while children experience seven to 10 such episodes annually [5]. Recent evidence suggests that 64.1% of adults (18-65 years), 25.5% of children (<18 years), and 10.4% of elderly patients (>65 years) have cough complaints [6,7]. However, only less than 30% of these cough cases could be specified as productive or non-productive cough, while a majority of these cough cases remain unspecified (87.2% of pediatric, 71% of adults, and 66.4% of elderly patients) [6,7].

Acute cough is a leading symptom in exacerbations of asthma and chronic obstructive pulmonary disease, requiring healthcare utilization and adversely affecting quality of life [8]. It frequently leads to sleep disturbance, musculoskeletal pain, syncope, and occasionally, a more serious sequelae of foreign body aspiration or underlying cardiopulmonary disease [2,9]. Delayed or inaccurate diagnosis of acute cough etiologies in India is a significant issue that contributes to several negative outcomes, including missed detection of serious conditions such as pneumonia and tuberculosis, injudicious antibiotic use, and an increased risk of complications in vulnerable populations [2,9,10].

Cough sounds (such as “wet” versus “dry,” barking, staccato, or paroxysmal quality) and auscultations (wheeze or differential breathing sounds) can provide diagnostic clues for conditions such as COVID-19, pneumonia, asthma, and pertussis [11-14]. Cough sounds encompassing the entire spectrum of auditory characteristics discernible through direct listening to a patient’s cough, including its quality, timbre, duration, and associated features, alongside auscultatory findings obtained through stethoscope examination, play a pivotal role in the characterization and clinical assessment of cough [11,15,16]. The teaching at the medical colleges includes exhaustive training on auscultation, which includes auscultation of the lungs and hearing the sounds. This forms the primary basis of clinical examination of any patient. In routine practice, evaluation of acute cough relies heavily on clinical history, physical examination, including listening to cough sounds and chest auscultation, and basic investigations such as chest radiography [9]. However, overlapping symptoms, limited access to point-of-care diagnostics in Indian settings, and heterogeneity in clinician approaches contribute to variability in diagnostic accuracy and management decisions for both productive and non-productive cough [6,7,17-19]. The present study aimed to assess the use of cough sounds and auscultations alongside different diagnostic approaches for the evaluation and categorization of acute cough and compare the same with different healthcare professional (HCP) specialties. This will further help to guide the general practitioners to adopt a simple symptom-based approach to evaluate and manage the acute cough.

## Materials and methods

Study design and setting

This retrospective, cross-sectional, observational study analyzed all the electronic medical record (EMR) data available on the platform from the Indian patients presenting with acute cough between January 2017 and December 2023. Patients were categorized as pediatric (<18 years), adult (18-65 years), and elderly (>65 years). Patients with productive cough included all those who complained of cough with sputum, wet cough, cough with expectoration, cough with running nose, purulent cough, or cough with mucus. Patients with complaints of dry cough, unproductive cough, allergic cough, cough with coryza, cough without expectoration, spasmodic cough, dry cough with rhinitis, cough without sputum, or cough without bronchospasm were classified as patients with nonproductive cough. Patients who did not have the cough type mentioned as “wet” or “dry” or using any of the abovementioned terminologies on the EMR were considered as patients with unspecified cough. The data were captured by the EMR tool provided by HealthPlix (Bengaluru, India) to the practicing doctors who opted to use it. The cough characteristics (e.g., duration, frequency, nature, and associated complications) were captured as mentioned in the EMR. The majority of the doctors who used this tool belonged to urban settings. The EMR database was generated through routine clinical documentation entered by treating physicians into a standardized EMR system available at their centers. The EMR system is maintained by the respective clinics and healthcare institutions, with data storage on secure, password-protected institutional servers in compliance with applicable data protection and confidentiality regulations. For the present analysis, anonymized data were extracted by authorized personnel following institutional approvals. Access to the database was restricted to the study investigators and was granted solely for research purposes. The results on prevalence, clinical characteristics, treatment, and prescription patterns have already been published [6,7,18,19]. The study was conducted in accordance with the International Council for Harmonization of Technical Requirements for Pharmaceuticals for Human Use (ICH) guidelines; the study protocol was reviewed and approved by the Royal Pune Independent Ethics Committee (EC) (approval number: RIPEC121123 dated 8 November 2023). As this was a retrospective analysis of the EMR data, registration on CTRI was not mandatory. As the aggregated data was captured without any patient identifier, there was no need for informed consent.

The present study is a nested study that aims to evaluate cough sounds and auscultatory patterns, and identify the common diagnostic investigations used by different medical specialties for evaluating acute cough and categorizing it according to the cough-related complications reported during clinical consultations.

Statistical analysis

A convenience sampling method was used, and there was no sample size calculation as the data were from the retrospective cross-sectional analysis of available EMR data. Statistical analysis for this study was carried out using Stata version 15.1 SE (StataCorp LLC, College Station, TX). Categorical data were summarized by age groups and doctor specialty using frequency (n) and percentages. Comparative analysis was done using the percentage occurrence of the events among the groups.

## Results

Prevalence of acute cough

A total of 1,250,846 (55.55%) patients with acute cough were analyzed from the overall database of 2,251,735 patients. The majority (i.e., 904,017 (72.27%)) were adults, followed by elderly patients (201,529, 16.11%) and pediatric patients (145,300, 11.62%). Unspecified cough was most reported across all age groups - pediatric: 128,184 (88.22%); adult: 707,788 (78.29%); and elderly: 154,129 (76.48%). Non-productive cough was predominant in pediatric (10,960, 7.54%) and adult (105,205, 11.64%) patients, whereas productive cough was more commonly reported in elderly patients (26,656, 13.22%) (Table 1).

**Table 1:** Distribution of acute cough across age groups n, number of patients; %, percentage of patients Note: For cough, the percentage of patients was calculated based on the total number of patients (N = 1,250,846). For the types of cough, the percentage was determined on the basis of the number of patients in each age group (pediatric, n = 145,300; adult, n = 904,017; elderly, n = 201,529).

Age group	Type of cough	n (%)
Pediatric (<18 years)	Cough	145,300 (11.62)
	Productive	6,156 (4.24)
	Non-productive	10,960 (7.54)
	Unspecified	128,184 (88.22)
Adult (18-65 years)	Cough	904,017 (72.27)
	Productive	91,024 (10.07)
	Non-productive	105,205 (11.64)
	Unspecified	707,788 (78.29)
Elderly (>65 years)	Cough	201,529 (16.11)
	Productive	26,656 (13.22)
	Non-productive	20,744 (10.29)
	Unspecified	154,129 (76.48)

Comparative analysis of the auscultation patterns

**Figure 1:**
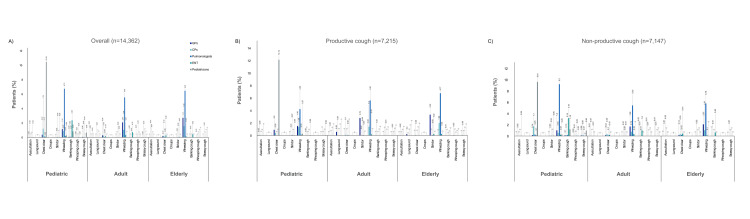
Comparative analysis of the auscultation patterns (A) Overall auscultation patterns. (B) Auscultation patterns in patients with productive cough. (C) Auscultation patterns in patients with non-productive cough.

Age and specialty exhibited distinct patterns for patients with acute cough. Wheezing was the most prominent clinical finding across all ages, most reported by pulmonologists: 376 (6.73%) cases in pediatric patients, 2,954 (5.53%) in adults, and 934 (6.45%) in the elderly. Pediatricians indicated the largest proportion of patients were with a clear chest (1,654, 10.44%), whereas otorhinolaryngology (ENT) specialists identified barking cough in both pediatric (61, 2.36%) and adult (98, 0.72%) patients. Stridor remained negligible across all ages and specialties (Figure 1A). In productive cough, pulmonologists most frequently reported abnormal lung sounds across age groups, wheezing in 117 (4.22%) of pediatric, 1,714 (5.61%) of adult, and 659 (6.77%) of elderly patients. Pediatricians most documented clear chest findings in the pediatric population (637, 12.13%). Stridor was rare, only five (0.05%) by pulmonologists and one (0.22%) by ENT specialists in elderly patients (Figure 1B). In non-productive cough, wheezing was commonly reported by pulmonologists in 259 (9.20%) pediatric patients, 1,240 (5.43%) adults, and 275 (5.78%) elderly patients, respectively. Pediatricians reported clear chest in 1,017 (9.61%) of pediatric patients, while ENT specialists noted barking cough in 61 (3.16%) pediatric patients and 98 (1.00%) adults; stridor was uncommon across age groups and specialties (Figure 1C).

Diagnostic approaches in the evaluation of acute cough

**Figure 2:**
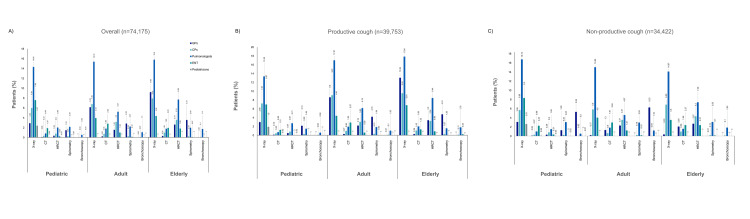
Diagnostic approaches in the evaluation of acute cough (A) Diagnostic approaches in the evaluation of acute cough overall. (2) Diagnostic approaches in the evaluation of productive cough. (C) Diagnostic approaches in the evaluation of non-productive cough.

Chest X-ray was the most frequently recommended diagnostic test ordered by HCPs irrespective of the specialty across all age groups - 3,767 (15.7%) pediatric patients, 33,850 (34.9%) adults, and 7,869 (56.4%) elderly. Very few patients underwent spirometry: 561 (2.33%) pediatric patients, 4,716 (4.86%) adults, and 919 (6.59%) elderly. High-resolution computed tomography (HRCT) was done in 415 (1.73%) pediatric patients, 12,988 (13.39%) adults, and 3,390 (24.31%) elderly, while computed tomography (CT) was done in 209 (0.87%) pediatric patients, 3,534 (3.64%) adults, and 830 (5.95%) elderly. Bronchoscopy was done in 42 (0.17%) pediatric patients, 772 (0.8%) adults, and 312 (2.24%) elderly. Pulmonologists and ENT specialists frequently ordered CT and HRCT, while spirometry was frequently ordered by pulmonologists and general physicians (GPs), and bronchoscopy by pulmonologists and GPs, irrespective of age (Figure 2A).

Investigation patterns in productive cough

Chest X-ray was the most utilized diagnostic tool across age groups, predominantly recommended by pulmonologists, followed by GPs across the patient age groups. In pediatric patients, pulmonologists, consulting physicians (CPs), and ENT specialists used radiography more than spirometry, HRCT, CT, and bronchoscopy. In adults, pulmonologists recommended chest X-ray and HRCT. The use of bronchoscopy, usually advised by pulmonologists, was minimal (Figure 2B).

Investigation patterns in non-productive cough

Chest X-ray was the primary diagnostic tool and was frequently ordered, while GPs and CPs recommended it mostly for the elderly. Pediatric evaluations showed conservative imaging use. Advanced imaging was used selectively: pulmonologists preferred HRCT, while ENT specialists occasionally used CT and HRCT. Spirometry was moderately used by pulmonologists and understandably less frequently by GPs. Bronchoscopy remained the least utilized test across all age groups and was reserved mainly for complex diagnostic cases under pulmonology care (Figure 2C).

Complications related to acute cough

**Figure 3:**
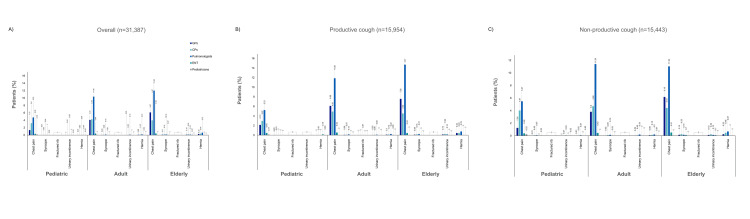
Complications related to acute cough (A) Complications related to acute cough overall. (B) Complications related to productive cough. (C) Complications related to non-productive cough.

Chest pain was the predominant complication of acute cough for which most patients visited across specialties and age groups - 1,631 (6.78%) pediatric patients, 22,906 (23.61%) adults, and 5,095 (36.58%) elderly. Patients with syncope, hernia, and urinary incontinence were comparatively uncommon (Figure 3A).

In patients with productive cough, chest pain was the most common complication for seeking medical care across specialties and age groups. Other complaints, such as syncope, hernia, and urinary incontinence, were infrequent. Consultations with the pediatrician and ENT (across all age groups) for the cough-related complications were negligible (Figure 3B).

In patients with non-productive or dry cough, chest pain was the most frequently reported complication across all age groups, particularly those visiting pulmonologists. Syncope, hernia, and urinary incontinence were comparatively uncommon across ages and specialties. ENT consultations were minimal for the complications, irrespective of age (Figure 3C).

## Discussion

Cough is a very common symptom that necessitates attention. While cough is studied in detail, the real-world data on acute cough in India is limited. The present study used the huge EMR database and highlighted the evolution of acute cough assessment post-pandemic and how the clinical evaluation is becoming increasingly sparse at the primary care level. The results on the prevalence, clinical characteristics, treatment, and prescription patterns for the management of acute cough captured through this database have already been published earlier [6,7,18,19]. Our research elucidates heterogeneity in reporting cough sounds, auscultatory patterns, and diagnostic decision-making, demonstrating the multifactorial nature of acute cough alongside the significance of patient characteristics and physician expertise. Listening to cough sounds and auscultations are important in the routine assessment of acute cough. The cough sounds, auscultatory profile, and diagnostic practices varied systematically by specialty and age, with pulmonologists demonstrating greater reliance on imaging and functional testing. In our study, chest X-ray was one of the most widely employed imaging tools for the diagnosis of diverse thoracic conditions, including cough, which is in line with the earlier investigations [2,20]. In addition, chest pain is presented as the most prominent cough-related complication. Furthermore, auscultation profiles varied distinctly across age groups and specialties based on cough type. Wheezing was more frequently reported to pulmonologists, particularly among adults and elderly patients, while ENT physicians predominantly documented barking cough, especially in younger populations, indicative of productive cough with clear chest.

Detailed auditory and auscultatory assessment of cough formed a cornerstone of respiratory examination, with experienced clinicians to distinguish between productive and non-productive coughs and to recognize the barking quality of croup, the whooping character of pertussis, or the honking nature of psychogenic cough [2,12,21,22]. This broader acoustic and auscultatory signature can provide immediate diagnostic clues that guide appropriate management pathways. Cough sounds carry vital information about the respiratory system and the associated pathologies, with strong correlation to airflow characteristics, including forced expiratory volume in 1 s and forced vital capacity ratios [23]. More recently, automated cough analysis has demonstrated adequate diagnostic accuracy in detecting common childhood respiratory diseases, including pneumonia, bronchiolitis, and upper and lower respiratory tract disorders without requiring clinical examination or supplemental investigations [24]. A critical gap exists in contemporary primary care practice, wherein GPs report poor understanding of screening and cough categorization, with only a minority expressing confidence in their ability to evaluate and decipher them [17,22]. This study highlights the need to develop simple, easy-to-use applications using artificial intelligence to analyze cough sounds and guide the probable diagnosis. Such applications can be handy for the primary care practitioners and will provide valuable information on the disease progress, and will also help avoid unnecessary investigations.

Our study identified distinct diagnostic approaches between cough types. GPs predominantly used imaging over systematic cough sound evaluation, with chest X-ray most frequently employed across all groups and cough types, consistent with guidelines for assessing conditions with lower-airway involvement, such as pneumonia [1,2,25]. Advanced imaging and functional tests (HRCT, CT, spirometry) were used for evaluation of persistent or complicated wet cough or to examine the underlying airway pathology, such as asthma, interstitial lung disease, or other non-infectious etiologies in dry cough. However, imaging use was relatively conservative in pediatric populations due to numerous challenges, including recommendations to limit radiographic exposure [26]. Bronchoscopy was rarely used in either type of cough; this is in line with its reserved role for patients with red-flag features or unclear etiologies [1]. Collectively, these findings reveal that cough sounds and auscultatory profiles are minimally utilized for diagnosis, highlighting the need for enhanced GP training in cough sound evaluation. This is in line with an earlier assessment of family physicians’ confidence in managing cough, which revealed notable uncertainty, highlighting the need for targeted education to reinforce, validate, and strengthen GPs’ knowledge and decision-making regarding effective cough management strategies in clinical practice [17,22]. In India, a substantial proportion of the population seeks healthcare in resource-constrained settings where advanced diagnostic facilities may not be readily available. In such contexts, it is imperative that primary care physicians are adequately trained to rely on fundamental clinical skills, particularly thorough history-taking regarding cough characteristics and meticulous chest auscultation, to effectively evaluate and manage patients. Training programs should emphasize these core competencies to ensure quality care delivery even in resource-limited environments [17,22].

The expanding use of diagnostic technologies has contributed to increased reliance on investigations, potentially diminishing emphasis on essential clinical examination skills. Concurrent time constraints in primary care and insufficient structured instruction in cough sound and auscultatory pattern recognition may further limit clinician confidence in cough assessment [11,27]. Evidence indicates that targeted respiratory sound training enhances diagnostic accuracy; however, it remains inconsistently incorporated into general practice curricula [28,29].

Early recognition of red flags and complications by GPs is essential for appropriate patient management. The present study identified chest pain as the most common complication in both productive and non-productive cough presentations, but its prevalence varied depending on the age of the patient and the specialty visit, with most patients requiring pulmonology consultation. This finding corroborates prior evidence documenting chest pain as one of the most common adverse events associated with prolonged cough [2,8]. However, it also highlights the lack of GP consultation for symptom-based assessment of acute cough, especially non-productive cough. Other complications, such as syncope, hernia, and urinary incontinence, were less commonly reported. Persistent severe coughing can result in complications such as vomiting, musculoskeletal pain, urinary incontinence, syncope, and depression, further emphasizing the critical role of GPs in early detection and management [8].

Our findings support minimizing unnecessary diagnostic testing of acute cough by promoting evidence-based clinical decisions and acoustic examination skills (especially for GPs) [17,30]. Careful auscultation of cough sounds provides valuable diagnostic information without additional cost or radiation exposure [16]. Besides, incidental findings may generate patient anxiety, trigger expensive additional investigations, and create psychological distress from false-positive results, despite their clinical significance in imaging studies [31,32]. In addition, excessive investigation of acute cough in its early stages may contribute to inappropriate antibiotic prescriptions, escalate healthcare expenditures, and result in overutilization of imaging resources [30,33]. Acoustic cough analysis can help in distinguishing cough types and detecting underlying pathology [34]. Furthermore, emerging digital technologies and artificial intelligence algorithms can further improve objective cough sound analysis; these technological advances can offer promising tools for non-invasive cough assessment [16,35]. There are multiple software applications available and many in the pipeline to assess the cough sounds. We need to have these tested in the Indian population to provide a meaningful tool for acute cough assessment and make it a part of routine practice.

The present study demonstrates several notable strengths; the analysis of over 22,50,000 patient records provides robust statistical power and a comprehensive representation of real-world clinical practice across India. By incorporating data from five distinct medical specialties: GPs, CPs, pulmonologists, pediatricians, and otorhinolaryngologists (ENT), the research offers valuable insights into specialty-specific approaches to cough diagnosis and management. However, important limitations warrant consideration. The lack of follow-up data represents a significant constraint, as the study did not capture treatment outcomes, disease progression, or long-term patient trajectories, limiting assessment of clinical effectiveness and prognosis. Considering the retrospective nature of the analysis, there is a possibility of missing data, and the assessment of cough sounds not being the priority is not recorded on the EMR, hence significant under-reporting as seen in the study results. The study also revealed a high proportion of unspecified cough, which is consistent with the practice at the primary care level. Being retrospective data, there is a potential underreporting of auscultation findings. There is a selection bias toward urban EMR users, and hence, results cannot be extrapolated to rural settings. It should also be noted that the COVID-19 pandemic substantially altered acute cough presentations, evaluation, and documentation. Pre-pandemic patterns showed stable consultations, with several cases recorded as unspecified. During the pandemic, consultations increased, nonproductive cough became more prevalent among adults and pediatric age groups, while productive cough was more prominent among the elderly, and documentation gaps widened due to diagnostic pressures and COVID-19 symptom overlap. After the pandemic, acute cough prevalence rose further, predominantly unspecified cases, highlighting ongoing respiratory effects and the need for improved clinical documentation and categorization [36]. The study period overlap of the current research with the COVID-19 era from 2020 to 2023 may have substantially influenced clinical presentations, healthcare-seeking behavior, diagnostic practices, and documentation patterns, though this impact was not specifically analyzed or controlled for in the study design.

## Conclusions

This study revealed distinct clinical profiles between productive and non-productive acute cough and provided insights into the acute cough evaluation practice in India. Use of cough sound and auscultatory pattern for acute cough evaluation by GPs may offer a cost-effective, non-invasive approach for accurate disease diagnosis. Acoustic and auscultatory assessment can minimize unnecessary testing while optimizing resources through targeted, minimal-ingredient treatments. Future research should develop standardized classification tools and age-specific algorithms to enhance early diagnosis and better patient outcomes in primary care settings.
